# The New Old CD8+ T Cells in the Immune Paradox of Pregnancy

**DOI:** 10.3389/fimmu.2021.765730

**Published:** 2021-11-16

**Authors:** Lilja Hardardottir, Maria Victoria Bazzano, Laura Glau, Luca Gattinoni, Angela Köninger, Eva Tolosa, Maria Emilia Solano

**Affiliations:** ^1^ Laboratory for Translational Perinatology- Focus: Immunology, University Department of Obstetrics and Gynecology, University Hospital Regensburg, Regensburg, Germany; ^2^ Department of Immunology, University Medical Center Hamburg-Eppendorf, Hamburg, Germany; ^3^ Department of Functional Immune Cell Modulation, Regensburg Center for Interventional Immunology, Regensburg, Germany; ^4^ University of Regensburg, Regensburg, Germany; ^5^ Department of Obstetrics and Gynecology of the University of Regensburg at the St. Hedwig Hospital of the Order of St. John, Regensburg, Germany

**Keywords:** fetal tolerance, feto-maternal interface, decidual CD8+T cells, immune tolerace, Immune regulation, pregnancy, exhaustion

## Abstract

CD8+ T cells are the most frequent T cell population in the immune cell compartment at the feto-maternal interface. Due to their cytotoxic potential, the presence of CD8+ T cells in the immune privileged pregnant uterus has raised considerable interest. Here, we review our current understanding of CD8+ T cell biology in the uterus of pregnant women and discuss this knowledge in relation to a recently published immune cell Atlas of human decidua. We describe how the expansion of CD8+ T cells with an effector memory phenotype often presenting markers of exhaustion is critical for a successful pregnancy, and host defense towards pathogens. Moreover, we review new evidence on the presence of long-lasting immunological memory to former pregnancies and discuss its impact on prospective pregnancy outcomes. The formation of fetal-specific memory CD8+ T cell subests in the uterus, in particular of tissue resident, and stem cell memory cells requires further investigation, but promises interesting results to come. Advancing the knowledge of CD8+ T cell biology in the pregnant uterus will be pivotal for understanding not only tissue-specific immune tolerance but also the etiology of complications during pregnancy, thus enabling preventive or therapeutic interventions in the future.

## Introduction

Sir Peter Medawar, a pioneer in transplantation biology, was the first to identify the immunological paradox of pregnancy. By formulating the research question 60 years ago “how does the pregnant mother contrive to nourish within itself, for many weeks or months, a fetus that is an antigenically foreign body?’’, he set the ground for the field of reproductive immunology. In pregnancy, the fetus remains protected from maternal immune responses as the multilayered placental villi acts as a semi-permeable barrier for the bi-directional migration of immune cells from fetal and maternal blood (1). However, the semi-allogenic placental trophoblast cells are in direct contact with the leucocyte-rich uterine mucosa, referred to as decidua. Medawar’s hypotheses to solve this paradox included the anatomical separation between the mother and the fetus, fetal antigenic immaturity and the immunological indolence of the mother (2, 3). Although these hypotheses have been tested across the years, this question remains incompletely answered, and investigation on this complex topic is still ongoing. As yet, a fine tuning of uterine leucocyte function has become evident. Systemic changes synergize with the intra-uterine microenvironment to shift the differentiation of antigen presenting cells (e.g. macrophages and dendritic cells) towards tolerogenic responses that promote the accumulation of regulatory T cells while restricting local cell activation and cytotoxicity. Of particular abundance in the early pregnant uterus, NK cells are critical for vascular changes and trophoblast invasion in early pregnancy (4). Less well understood to date is the role of CD8+ T cells. Due to their cytotoxic nature towards allogenic cells, the presence of uterine CD8+ T cells is of high interest in the context of fetal immune tolerance. In organ transplantation, CD8+ T cells are important effectors mediating allograft tissue damage. By contrast, in the pregnant uterus, CD8+ T cells despite being the most abundant T cell subset do not trigger lytic responses towards the allogenic placental trophoblast. Within the nonpregnant human uterus, CD8+ T cells have cytotoxic capabilities with fluctuating activity through the menstrual cycle (5). In first trimester pregnancy, T cells constitute approximately 5-20% of total decidual leucocytes of which CD8+ T cells are approximately 45% (6, 7). Although decidual NK cells have received great attention, their frequencies remain stable throughout pregnancy, whereas T cells actively increase (8–10). In the past few years, studies on decidual CD8+ T cells have kept pace as new findings on CD8+ T cell properties are emerging, which characterize CD8+ T cells by e.g. expression of functional receptors, secretion profile and antigen specificity. Due to the heterogeneity of CD8+ T cells and current methodological approaches, classification of CD8+ T cell subsets remains a dilemma as controversy colors the scientific community within CD8+ T cell biology (11–13). With that in mind, in this review, we aim to provide an overview of the phenotypes and function of CD8+ T cells in the pregnant uterus. In particular, we seek to highlight recent findings of human decidual CD8+ T cells and their role in pregnancy, including their contribution to the immunological paradox of pregnancy. When required, we will refer to translational aspects, for example in mouse models. We will focus on conventional CD8+ T cells, which express the ɑ and β chains of the T cell receptor (TCR), although the decidua contains also unconventional CD8+ T cells, such as those expressing the γ and δ chains of the TCR (14). We will discuss the most relevant issues limiting our understanding of CD8+ T cell biology in the context of pregnancy and highlight the gaps in knowledge that are required to be bridged, in order to advance this field. In general, investigations of the decidual CD8+ T cell compartment have largely complied with a selection of methods and markers (**Supplementary Table 1**, **Supplementary Figure 1**) that allows for a relatively unifying interpretation of their differentiation profile. In order to compare this information about decidual CD8+ T cell subsets, we re-analysed data on first trimester CD8+ T cell single cell transcriptome, published by Chen et al. (15) and report the findings about equivalent populations that are described in the literature.

## CD8+ T Cell Subsets and Differentiation Trajectories in Decidua

T cells are atypical somatic cells as after enduring quiescence for years, they can initiate massive proliferation and differentiation, resulting in multitude of heterogeneous cell subpopulations with diverse properties (16). **Supplementary Table 1** and **Supplementary Figure 1** contain information on the different CD8+ T cell subsets with a breakdown of CD8+ T cell subsets, their abbreviation, and cell-specific markers listed throughout the manuscript. The differentiation profile of CD8+ T cells populating the decidua (**Figure 3**) differs largely from that in peripheral blood (**Table 1**), indicating a highly tuned homeostasis of decidual CD8+ T cells.

**Table 1:** CD8+ memory populations in human decidua.

	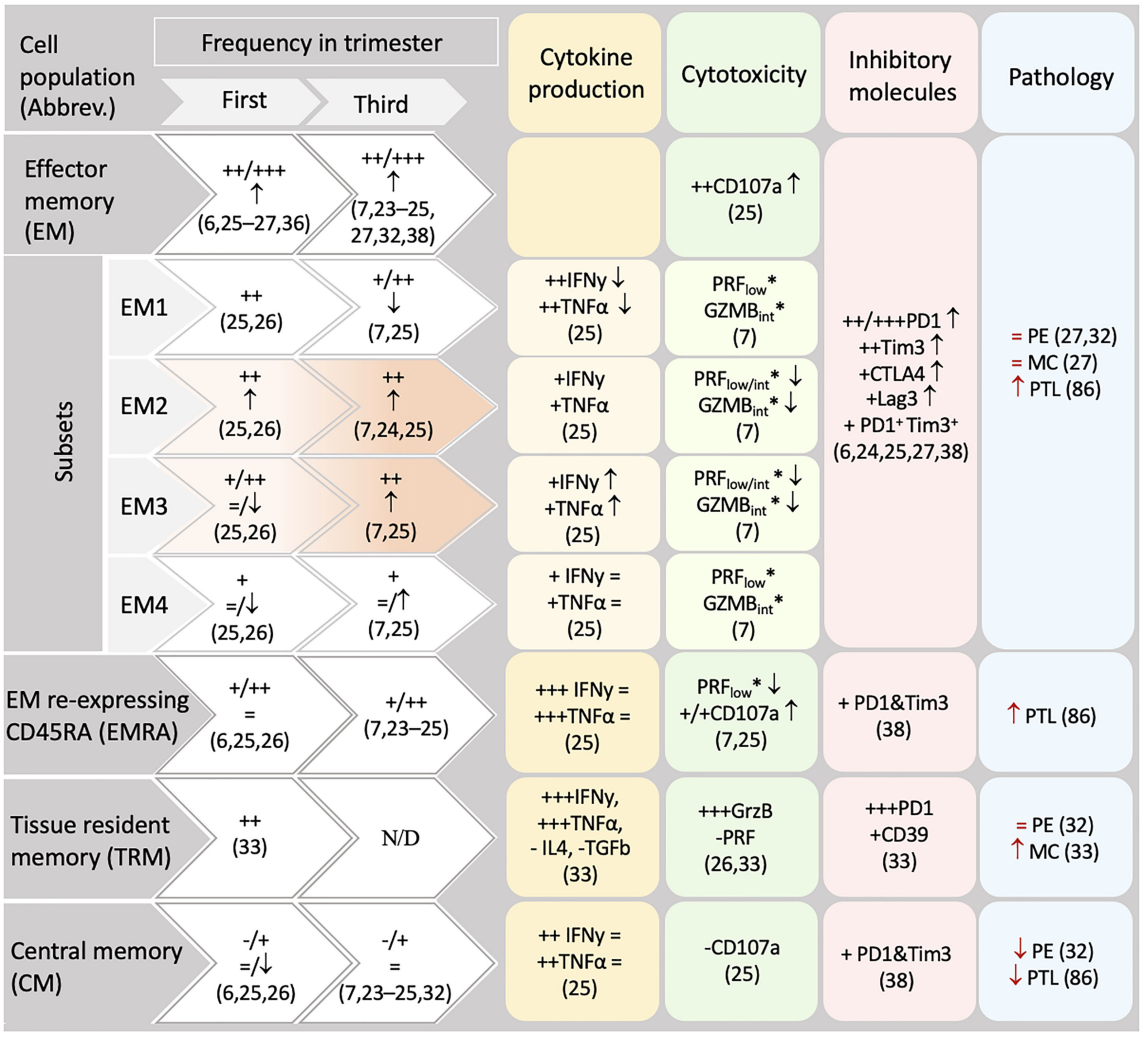	

The comparison is based on published flow cytometry analysis using markers CD45RA/RO and CCR7 as well as inhibitory markers and cytokine expression that allow for direct comparison. No data from second trimester was available fulfilling these criteria. Increased intensity of an orange background in EM2 and EM3 populations indicates their significant increase from first to third trimester. Black symbols **↑**, **↓,** and **=** indicate significantly higher, lower or not significantly different compared to CD8+ T cells in peripheral blood. **-** very low (0-5%); + low: 6-20%; ++ high: 21-55%; +++ very high >56% of the reference population. The symbol “/” represents diverging values. N/D: data not determined. Red symbols **↑, ↓, =** show populations significantly increased, significantly decreased, and not significantly different, respectively in a named pathology compared to normal pregnancy. Cytokine secretion/cytoxicity was measured following PMA/Iomycin stimulation except those marked with “*”. PRF, perforin-1; GrzB, granzyme B; PE, preeclampsia; PTL, pretermlabor; MC, miscarriage.

**Figure 1:**
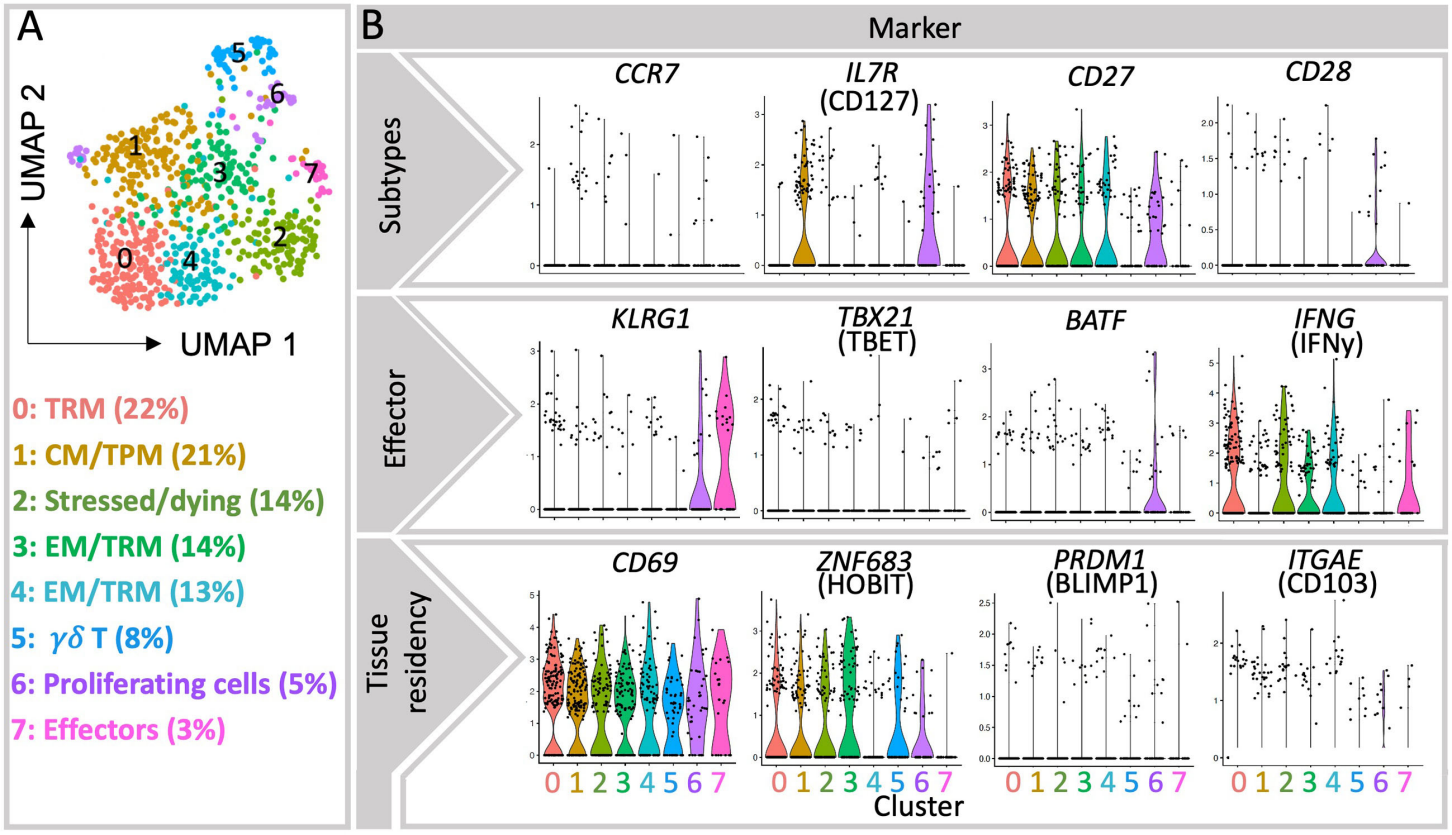
Single-cell transcriptomic analysis of decidual CD8+ T cells from publicly available database. **(A)** UMAP visualization of ∼735 live MS4A7- CD3E+ CD8A+ single cell transcriptomes obtained from the decidua of three healthy first trimester pregnancy samples (15). Each symbol (circle) represents a cell, and the colors indicate the eight clusters identified by Seurat. **(B)** Violin plots show the gene expression of selected genes in each cluster, particularly of TRM-associated transcription factors Hobit and Blimp1, as well as Tbet and BATF, associated with effector differentiation (11). More detailed information in the expression of relevant markers is provided as heatmaps (**Supplementary Figure 2**).

According to the “circular” or “effector first” model, T cells undergo a “naïve->effector->memory” differentiation hierarchy. This model indicates that cells activated by a primary antigen exposure differentiate towards cytotoxic effector cells. Upon antigen clearance, a large subset of effector cells undergo apoptosis/clonal deletion (short-lived effector cells, SLEC), whereas a small fraction (memory precursors effector cell, MPEC) survive, give rise to long lived memory cells (17) (**Supplementary Figure 1**). This proposed lineage relationships is currently under debate, and the so-called “linear model”, or “memory first model” places memory cells as an intermediate stage in T cell development, proposing that naïve cells can directly develop into memory cells, some of which later develop effector function (18–22). In first and third trimester decidua the frequency of **naïve** cells within CD8+ T cells is generally lower than in peripheral blood (6, 7, 23–27) as expected in a peripheral tissue (**Supplementary Table 2**). Limited information on effector subsets is available due to the scarce use of the markers CD127 and KLRG1 that could serve to discriminate MPEC from SLEC in the immune phenotyping of decidual T cells (28). Our analysis of single cell sequencing data pinpoints that the small cluster (3%) number 7 includes SLEC (**Figure 1**), as indicated by the negativity for CD127, and the expression of KLRG1, granzyme H, B, perforin 1, CD160 and additional killer cell lectin-like receptors. Whether these cells are recently activated in the context of the current pregnancy or recruited to the uterus after previous antigen exposure i.e. in secondary lymphoid organs remains unclear. Bridging these gaps in knowledge could further elucidate important steps in the immune responses towards fetal/placental antigens in pregnancy.

**Figure 2:**
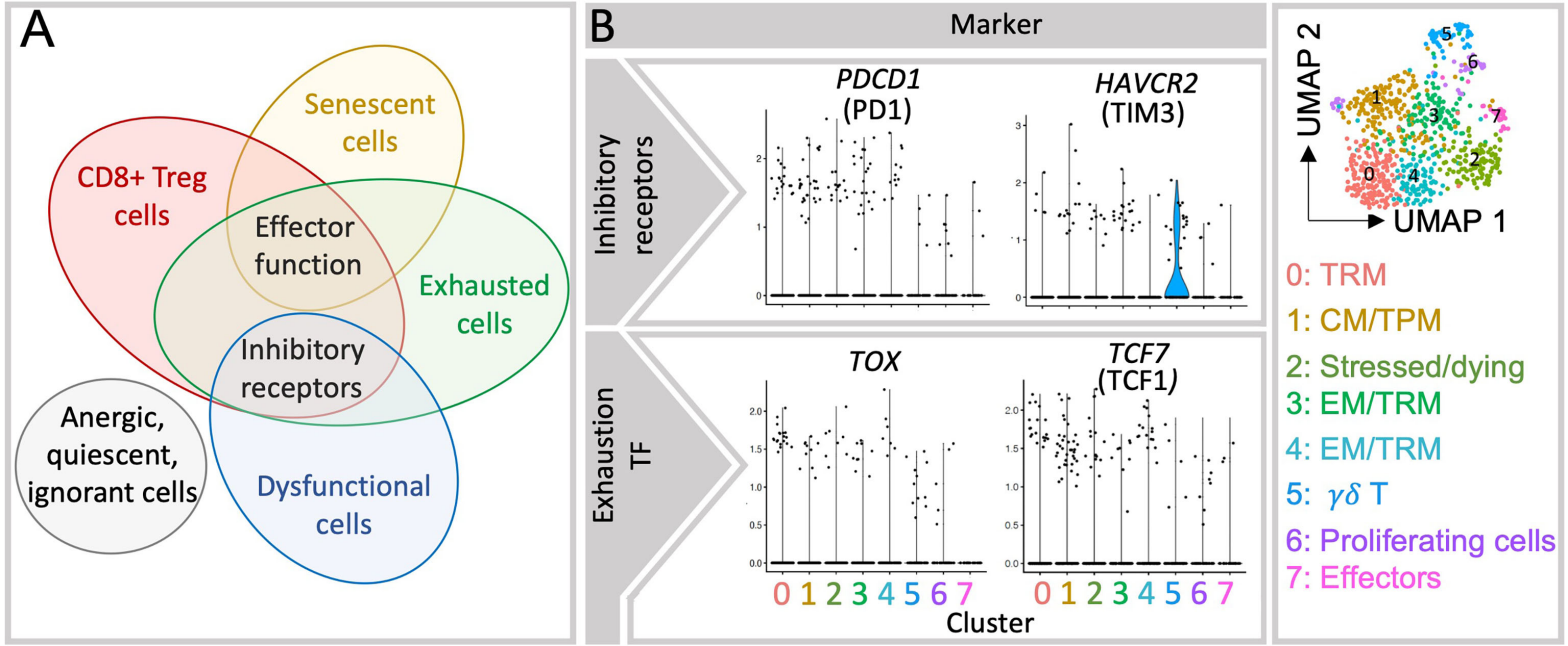
Currently reported decidual CD8+ T cell subsets present overlapping features. **(A)** Expression of inhibitory molecules is present in multiple cell populations in the decidua and appears insufficient to determine whether a given cell is exhausted, dysfunctional or has regulatory capabilities. Inhibitory receptors are co-inhibitory molecules that hinder T cell activation and functions, those include i.e. PD1, CTLA4, Tim3 (32). Effector function applies to activate CD8+ T cells that is mediated with i.e. secretion of effector cytokines and/or cytolytic molecules in order to resolve a given threat. **(B)** Violin plots show the gene expression of inhibitory receptors (PD1 and Tim3) and transcription factors associated with exhausted cells (TOX and TCF1) in each cluster, which fall in many of the clusters shown in the UMAP plot (right). TF, transcription factors.

More data is available on the heterogeneous memory CD8+ T cells subsets within human decidua, as summarized in **Table 1**. After responding to their cognate antigen, memory CD8+ T cells acquire distinct phenotypes and migration potential and endure long-term. They can quickly respond to re-stimulation by vigorous proliferation, cytotoxicity and secretion of effector cytokines (11). Compared to other memory CD8+ T cells, the stem cell memory (SCM) subset represents a naïve-like memory cell population with enhanced capacity to self-renew that serves as a precursor of other memory cell subsets (29). Due to the massive expansion of memory T cells across pregnancy and the long-term maintenance of robust T cell-memory to former pregnancies (30), it is tempting to hypothesize that fetal specific SCM are induced in pregnancy. To date it remains uninvestigated whether SCM cells are present in the decidua or more likely in uterus draining lymph nodes, as SCM are mainly found in lymphoid organs (31). A further elucidation of the transcriptional signature of this cell subset may allow its identification in the future, which is currently challenging e.g. in our single cell sequencing data analysis due to the limited number of cells analyzed and the low expression of key transcription factors. In this regard, within cluster 1, now defined as CM/TPM, we observed an enhanced expression of the mRNA encoding TCF-1 and CCR7, suggesting that, if present in the uterus, SCM could also cluster here (**Figures 1** and **2**).

The relative presence of two long-standing and divergent memory populations namely central memory (CM) and effector memory (EM) cells is well described in the uterus (6, 7, 23–27, 33). Due to expression of lymphoid homing receptors, CM cells are highly prevalent in secondary lymphoid organs and circulate through blood and lymph. They only account for a small fraction in decidua that ranges from 3-15% of CD8+ T cells in first and third trimesters as shown by flow cytometry data (6, 7, 23–26, 34, 35). Recently, an additional memory population capable of circulating in blood and lymph nodes but also of trafficking within peripheral tissues was identified and referred to as peripheral memory (Tpm) T cells (11, 36). Tpm cells display intermediate CX3CR1 expression, which has not yet been studied in the decidua and appears as a novel and important marker for distinguishing the distinct migratory patterns that define EM, CM and Tpm CD8+ T cells (11). Analysis of the existing scRNA sequencing data for decidual immune cells reveals a rather large cluster (21%) characterized by high levels of CD127 and CCR7, which could be indicative of CM-like and Tpm subsets, advocating a more prevalent population than shown by flow cytometry data in the pregnant uterus (**Figure 1**). As intermediate expression range of CX3CR1 was not detectable on mRNA level, we cannot separate CM and Tpm subsets. It remains to be elucidated whether these observations are due to a potential contamination with PBMCs or due to particular migration patterns of peripheral cells to the uterus during pregnancy.

EM cells, expressing receptors necessary for entering local inflammation sites, circulate through non-lymphoid tissues and exhibit higher cytolytic activity. It is not surprising that EM cells accumulate in the decidua (6, 7, 23–27) to account as the most prevalent CD8+ T cell population in first and third trimester, ranging from 48-83% of CD8+T cells (6, 7, 23–27, 33, 34, 37, 38). Decidual CD8+ EM T cells possess elevated expression of inhibitory receptors such as Programmed cell death protein 1 (PD1), T-cell immunoglobulin mucin family member 3 (Tim3), Cytotoxic T-lymphocyte-associated protein 4 (CTLA4), Lymphocyte activation gene 3 (Lag3) and CD39 compared to peripheral CD8+ T cells (6, 24, 25, 27, 39). Among the EM subset, the less differentiated EM1 population (CD27+CD28+) is of the highest frequency in the first trimester decidua (25, 26). In contrast, in the third trimester decidua, EM1 are outnumbered by more differentiated EM2 and EM3 (**Table 1**), whose frequency is significantly increased compared to first trimester (25). An additional terminally differentiated EM cell population defined by the re-expression of CD45RA are the so-called effector memory re-expressing CD45RA (EMRA) cells. EMRA cells are found in decidua, ranging from 11-45% (6, 7, 25, 26, 34, 38) among which the more differentiated phenotype (CD27-CD28-) is the most prevalent (26). Similarly to EM, cell surface membrane expression of inhibitory receptors such as PD1 and Tim3 have been detected on EMRA cells (39). The accumulation of particular subpopulations of EM and EMRA subsets may mirror a dynamic differentiation throughout pregnancy e.g. in response to increasing antigen levels. Notably, defining markers for EMRA (CD45RA+CCR7-CD127-), would also include effectors, which should be kept in mind when relying on such gating strategy (11).

Remarkably, this notion of a dominating EM CD8+T cell profile in the decidua is challenged by the recognition of tissue resident memory (TRM) subsets which share some markers with EM. TRM migrate to non-lymphoid tissues and enter specific differentiation programs under the influence of tissue specific signals. TRM cells infrequently re-enter the circulation and account therefore for the first line of defense within tissues upon re-encounter with antigens, by subsequent secretion of proinflammatory cytokines or lysis of infected cells (40). TRM can have overlapping properties with other CD8+T cells including effector functions and/or high levels of inhibitory receptors (12, 40) and it is still debated whether TRM cells are terminally differentiated or not (16). TRM CD8+ T cells have been recently identified in mucosa of the whole human reproductive tract (41, 42) and in the decidua (6, 24–26, 33, 34) by the single or co-expression of the mucosal/tissue retention markers CD103 and CD69 (11). Among decidual CD8+ T cells ~26% are CD69+CD103+, which likely include the true tissue resident memory cell population, whereas ~50% are CD69+CD103-. CD69+CD103+ TRM cells expressed significantly higher levels of the inhibitory molecules PD1 and CD39 (34). These observations invite to reevaluate the relative abundance of EM and TRM subsets in the decidua, their similarities as well as differences, as reports before the identification of TRM may have mistakenly referred to them as EM.

The single cell transcriptome analysis scattered four further clusters of CD8+ memory T cells. Among them the most abundant cluster 0 (22%) represents TRM cells (**Figure 1**), further supporting recent flow cytometry data on the high prevalence of TRM cells in the uterus. The TRM profile was evident by the high expression of mRNA coding for TRM markers CD69, CXCR6 and Hobit, although the markers CD103 and Blimp1 were not particularly co-expressed in this or other clusters. Markers such as Hobit, CD69, and IFNy are also abundant in Cluster 2, which defines a population of stressed and/or dying cells. Hence, Cluster 2 may also contain TRM cells, which are known to be extremely susceptible to ATP-induced cell death (43). In contrast, the clusters 3 and 4 presented mixed gene signatures of EM and TRM subsets (**Figure 1** and **Supplementary Figure 2**). Here the prevalence of EMRA cells could not be described since CD45RA cannot be determined at the mRNA level. Memory cells scattered in cluster 3 presented a pattern of regulatory/inhibitory and pro-angiogenic genes together with Hobit, whereas cluster 4 was enriched in markers for effector function and low in Hobit. These observations suggest that under the complex stimuli at the decidual microenvironment TRM and EM may gain similar functional signatures that complicate their discrimination.

Collectively, the continuous progress in research on differentiation, phenotypical and functional features of CD8+ T cell subsets (16) opens new questions on the CD8+ T cell compartment in the pregnant uterus. The current findings indicate that decidual CD8+ T cells are largely antigen-experienced cells, on a path of enhanced differentiation (24). The progress of pregnancy and likely the chronic exposure to allo-antigens favor the accumulation of EM cells with a highly differentiated phenotype, with high expression of inhibitory receptors, which however remain as EM3 rather than transitioning to EMRA. Many of these previously called EM and EMRA cells express markers for tissue residency, indicating that they are retained in the tissue, instead of recirculating to blood and that previous observations about decidual EM cells may apply to more recently identified TRM cells.

## Antigen Specificity of Decidual CD8+ T Cells

The accumulation of antigen experienced CD8+ T cells in the uterus opens questions about their antigen specificity. The clonal repertoire of the TCR of decidual CD8+ T cells indicated that clonally expanded EM CD8+ T cells were more abundant in the decidua than in blood (27, 44) although it could not be tested if an expansion occurred in response to fetal-derived antigens. Along with these findings, CD8+ EM T cells expanding clonally in decidua during healthy pregnancy expressed high levels of PD1 underpinning the importance of the modulation of CD8+ T cell responses to promote immune tolerance (27). In line with these observations, the detection of CD8+ T cells specific for peptides derived from the HY chromosome allowed to identify a local expansion of fetal (HY) specific CD8+ T cells particularly in the decidua of women carrying male but not female offspring (24). HY-specific T cells were enriched in EM and EMRA subsets expressing high levels of PD1 and CD69 indicating a highly differentiated and tissue resident-like profile, likely in response to local antigen stimulation (24). Also compared to blood, an enrichment of CD8+ T cell clones specific for the frequent human cytomegalovirus (HCMV) and Epstein Barr (EBV) viruses (38) has been reported, pinpointing an increased capacity of decidual CD8+ T cell to fight these maternal infections.

In conclusion, while particular clones of CD8+ T cells, e.g. specific for fetal and viral antigens, are enriched at the decidua, their function may be modulated by the expression of PD1 and other inhibitory receptors to promote fetal immune tolerance. Intriguingly, it is to date unclear whether these selected clones are specifically recruited to the uterus, or if they undergo clonal proliferation locally in an antigen specific or unspecific fashion (45).

## Cellular Functional/Responsive States

During T cell development in the thymus, the incomplete deletion of self-reactive T cells makes further peripheral tolerance checkpoints crucial (46). Those include the generation of distinct responsive states to a given stimulus, that is, its functional state which can confer tolerance in the case of quiescence, ignorance, anergy, exhaustion, dysfunction and senscence. Hence, CD8+ T cell fate could be considered to follow two parallel and overlapping developmental courses/trajectories, one being the differentiation of a naïve cell to a variety of effectors and memory cells, the other being the functional state of the cell. These functional states may hold relevance at the feto-maternal interface to support a successful pregnancy, as Sir Peter Medawar’s hypothesis about the anatomical separation between maternal immune cells and fetal cells has been refuted. Tolerance states can be recognized in diverse CD8+ T cell subsets, thus rather than discussing how these states are attained, we will revisit current information about their presence and importance in the decidua.

### Quiescence, Ignorance and Anergy

Quiescent and ignorant CD8+ T cells have in common that they are naïve T cells while anergic cells have previously encountered their respective antigen. While quiescence applies to all naïve T cells, regardless of their antigen specificity, ignorance applies to self-reactive T cells avoiding activation due to a low density of their cognate antigen or the antigen being spatially not available. In contrast, anergic CD8+ T cells have experienced a defective TCR stimulation due to the lack of co-stimulation during TCR activation (47). In the mouse endometrium, fluctuations in clonal anergy during the reproductive cycle support endometrial receptivity for implantation (48). During pregnancy, a role of T cell anergy at the feto-maternal interface is supported by the local lack of necessary T cell co-stimulators (48). Furthermore, hypo-responsiveness and ignorance contribute to CD8+ T cell tolerance to fetal antigens in mice (49–51). Taken together, although their extent in decidual CD8+ T cells has not been investigated in depth, the general tolerance mechanisms of quiescence, ignorance and anergy are likely to play a role at the feto-maternal tolerance.

### Exhaustion and Dysfunction

Following activation and differentiation into classical effector T cells, a cell can acquire an exhausted state if antigen stimulation persists chronically. Exhausted T cells can also arise early in infection, advocating that additional paths of development are also possible (52). Although there is no definitive marker to distinguish exhausted cells from other subsets (53) recent findings have pinpointed TOX as a key transcriptional regulator of T cell exhaustion. In chronic infection two populations of exhausted cells have been identified based on the expression of TCF1: one comprising TCF1^high^ stem cell-like progenitors that self-renew, give rise to and maintain the second population, a terminally differentiated exhausted T cell subset, which lack TCF1 expression (54). Besides that, exhaustion is often defined by the expression of a set of multiple inhibitory molecules, including PD1, Tim3, Lag3, Tigit, and CTLA4 (53). However, very high levels of inhibitory molecules suggest dysfunction rather than exhaustion (53). Important specific features of exhausted T cells are likely due to signals arising from the different microenvironments. Although in certain conditions exhausted T cells may retain the production of effector molecules, their atypical immune responses may prevent immunopathology in the host e.g. by control of infections (53, 55, 56). In other microenvironments, such as in tumors, exhausted T cells generally lack effector function and fail to control tumor growth acquiring a dysfunctional state (57). Importantly, although exhausted T cells may be dysfunctional, not all dysfunctional T cells are exhausted (53) and furthermore, dysfunction is as state that can arise from other cell fates, such as from anergy (25).

Despite the differences between the feto-maternal interface, chronic infections and tumors’ microenvironment, constant fetal antigen exposure in the decidua may trigger similar exhaustion signatures in CD8+ T cells. As summarized in **Table 1**, the expression of inhibitory receptors indicative of exhausted or dysfunctional states have been reported in diverse decidual CD8+ T cell subsets (6, 7, 24–27, 34, 37). A dynamic induction of exhaustion states may take place in the intrauterine environment, as PD1 and Tim3 expression increased in decidual CD8+ T cells through gestation (37). The majority of uterine exhausted-like cells gather within the EM compartment, although some were present within the CM and EMRA subsets (37). In contrast, the expression of TCF1 and TOX remains to be investigated in the decidua. So far, our analysis of single cell sequencing data showed that TOX is scarcely expressed in the EM and TRM clusters (**Figure 2** and **Supplementary Figure 2**). This expression did not coincide with the one of PD1 or Tim3 questioning the suitability of using these markers to define exhaustion (**Supplementary Figure 2**).

Taken together, difficulties in the identification and differentiation of exhausted and dysfunctional T cells have limited the progress in this field of research. The detection of exhausted cells by the expression of inhibitory markers may be misleading as various functional and competent CD8+ T cells, including effectors and regulatory cells, or dysfunctional cells express comparable sets of inhibitory molecules.

### Senescence and Deletional Tolerance

Senescence refers to a state in which highly differentiated CD8+ T cells have reached the end of their proliferative potential after repeated TCR stimulation but maintain their effector function (58, 59). Hence, senescence is more prevalent in advanced differentiation subsets, such as in EM3 cells (60), and generally correlates with cellular aging, partly due to reduced telomere length (59). Senescent CD8+ T cells are present in the human decidua, as identified by their positivity for CD57 and KLRG1 (37, 58). Because the presence of the CD57 epitope cannot be detected in transcriptome analysis, we could not verify these results in the transcriptome dataset. At last, CD8+ T cells can undergo deletional tolerance that removes the antigen specific expanded CD8+ T cell clones and favors the termination of the immune response (61). In mice, deletional tolerance takes place at the uterus-draining lymph nodes (50), thereby restraining antigen-specific responses at the feto-maternal interface. Therefore, both senescence and deletional tolerance appear as mechanisms at play in the pregnant uterus. However, their relative contribution to sustaining immune tolerance towards the fetus e.g. by limiting extensive proliferation of decidual CD8+ T cells specific for fetal antigens due to senescence or by reducing them by deletion during and after pregnancy (59) requires further investigations.

## Regulatory/Suppressive CD8+ T Cells

Decades before the discovery of the CD4+ Treg cells, CD8+ T cells embarked as the first T cell subset with a suppressive potential (62). However, difficulty in confidently distinguishing them phenotypically from other CD8+T cell subsets halted the progress of this research topic while research on CD4+ Treg cells bloomed following the identification of the transcription factor Foxp3 (63). In recent years, findings on suppressive CD8+ T cells, also called CD8+ Treg cells have underscored their importance in biological contexts such as in tumor tolerance, organ transplantation, autoimmune diseases in mice and humans (64–70). Yet, due to the overlapping characteristics with other effectors, memory and exhausted T cells populations, there is no general consensus about markers to unambiguously identify CD8+Treg cells (**Figure 2**). To date, CD8+ Treg cells have been identified based on the expression of FOXP3, the CD8aa receptor, the absence of CD28, and the specificity for hemoxygenase (HMOX)-1 derived peptides in humans (64, 71–75) as well as by CD122 positivity and restriction to Qa1 in mice. Inhibitory molecules, such as PD1, Tim3, CTLA4, Ly49, Lag3 and Tigit have also served to identify CD8+ Treg cells (63, 64). The expression of such inhibitory receptors was also described in CD8+HLA-DR+ cells with regulatory properties, whose enhanced positivity for IFNy and TNFα and degranulation ability appear related to induction of tolerance rather than exhaustion (76). Under the influence of environmental cues and of the particular immune phenomenon, diverse regulatory pathways were described for CD8+ Treg subsets (67) including suppression of proliferation of cytotoxic/autoreactive T cells, e.g. by triggering apoptosis of target cells, and by inhibition of their secretion of inflammatory cytokines. These actions may be mediated *via* cell-cell contact, suppression of costimulatory molecules in dendritic cells, Fas/FasL pathway, secretion of inflammatory (perforin 1, granzyme B (GrzB), IFNy) and/or immunosuppressive molecules (IL-10, TGFβ) (71). Due to the heterogeneity of the findings available, the great phenotypical and functional overlap of CD8+ Treg cells and conventional subsets (**Figure 2**) their classification and nomenclature as “regulatory” has been brought into question (71). However, the immune regulatory properties of CD8+ T cells in important biological phenomena cannot be denied.

To date, studies on decidual CD8+ T regulatory cells, as such, are scarce. In humans, the regulatory subsets CD8+HLA-DR+CD8+ T cells (76) and CD8+CD28- T cells (77–79) were enriched in decidua, compared to peripheral blood. Additionally, decidual CD8+ T cells expressing inhibitory receptors have been referred to as having a regulatory function in the decidua in mice and humans (39, 80) colliding with those studies that defined exhausted cells by the expression of the same inhibitory markers (e.g. PD1+ Tim3). In mouse pregnancy, a regulatory function for CD8+CD122+ T cells was evident as their adoptive transfer prevented inflammation-induced intrauterine growth restriction (IUGR) restoring placenta and fetal growth (72).

Taken together, the possibility of distinguishing CD8+ Treg from exhausted cells and other subsets is urgently needed for unifying the scientific community and the progress of the field of CD8+ T cell biology. Coupling an in-depth analysis of markers with functional assessment of candidate subpopulations may serve to dissect unambiguously the potential functional states and suppressive capabilities (25).

## CD8+ T Cell Function in Pregnancy

### Immune Tolerance and Homeostasis

Besides providing immunity towards pathogens invading this mucosal area, decidual CD8+ T cells execute tailored responses to the dynamic changes of the pregnant uterus (42). Intriguingly, CD8+ T cells are pivotal for immune tolerance and pregnancy success, as both a deregulation of CD8+ T cell recruitment and differentiation as well as their depletion in pregnancy can hamper immunotolerance and the success of pregnancy (72, 81–83). Hence, the frequency, differentiation, enrichment of markers for exhausted/regulatory function and particular cytokine secretion profile, allude to a tight regulation of the CD8+ T cell compartment at the intrauterine niche as a pivotal mechanism of immune homeostasis and tolerance towards the fetus, which are tilted in pathological contexts. In preeclampsia (35, 84), miscarriage (39, 80, 85) and intrauterine growth restriction (IUGR) (84, 86) increased CD8+ T cell counts have been observed at the feto-maternal interface. Additionally, in inflammatory conditions, such as miscarriage and preterm labor, the differentiation of decidual CD8+ T cells shifts towards a relative expansion of TRM in cases of miscarriages, as well as enhanced EM and EMRA in pregnancies undergoing preterm labor (**Table 1**) with detriment of CM subsets that appear reduced in both pathologies (87). The respective expansion of TRM and EM/EMRA CD8+ T cells with potent activation potential may in turn contribute to the pathophysiology of inflammation in these conditions.

Decidual CD8+ T cells express high levels of inhibitory receptors. Although it remains still unclear whether this reflects an exhausted state, this expression pattern can certainly influence CD8+ T cell activation to modulate their function (**Supplementary Table 3**). Concomitantly, the feto-maternal interface provides an environment rich in the respective ligands, pinpointing an important role for these immune checkpoint mechanisms in the success of pregnancy (**Supplementary Table 3**). In particular, PD1 in CD8+ T cells may bind programmed death-ligand 1 (PD-L1) at the feto-maternal interface (88) and inhibit their own T cell activation (89), alter the duration of the contact of CD8+ T cell with APC or target cell and enhance the proliferation of the PD1 expressing CD8+ T cells (90). Additional inhibitory functions can be mediated for example by blockage of antigen presentation in the case of Lag3, or of the interaction with costimulatory molecules in the case of CTLA4 and Tigit. The crosstalk of a selective combination of inhibitory receptors simultaneously expressed may further contribute to the unique function of CD8+ T cells in pregnancy. Particularly, co-expression of PD1 might modulate the function of Tim3 in pregnancy, that upon Gal9 binding drives to cell proliferation (39) rather than to the well described triggering of apoptosis in other contexts (91, 92). Such a modulation has been found in the tumor environment where PD1 reduces the Tim3/Gal9-mediated apoptosis (91). Also, CTLA4 synergizes with Tim3 pathways to promote anti-inflammatory cytokine release and healthy pregnancy (85). An additional player to consider in this context may be IFNy, that upregulates PD-L1 expression by trophoblast cells (93) and has also been shown to promote Gal9 expression (91).

In pregnancy complications, particular derangements within the CD8+ T cell compartment are observed. Indeed, while senescent CD8+ T cells are reduced in preterm labor accompanied by placental inflammation, exhausted cells are reduced in patients with placental inflammation at term labor (37). Also in preeclampsia, clonally expanded PD1+CD8+ EM are underrepresented (27), and exhausted decidual CD8+ T cells (co-expressing inhibitory receptors) diminished in women with recurrent miscarriage (39, 80, 94). In miscarriage, exhausted T cells additionally further displayed a deregulated transcriptome, secretion, and proliferation profile, which occurred together with enhanced clonal expansion of CD8+ EM cells, suggestive of a shift towards effector and inflammatory responses. The importance of inhibitory molecules was demonstrated experimentally by their blockade in mice, which resulted in miscarriage (39, 80, 94). Such plasticity for reversion of the exhausted state and regain of function of CD8+ T cells in intrauterine environment may be an important mechanism of host defense, for example in the case of intrauterine infections (37, 88), although it ultimately results in pregnancy pathology.

Functional features of the decidual CD8+ T cell have further been assembled by investigating their secretion profile (**Table 1**). Decidual CD8+ T cells are active producers of cytokines, and high levels of IFNy and TNFα were reported in decidual CM, EMRA, and EM CD8+ T cells (6, 25). Whilst these studies did not consider markers for tissue residency, a recent report indicated that TRM cells possess higher capacity than other CD8+ T cells to produce IFNy, TNFα, IL-4 and the regulatory cytokine TGFβ (34). In line with this, the single cell transcriptome analysis pointed to the TRM cell cluster as the one with highest IFNy mRNA levels (**Figure 1** and **Supplementary Figure 2**). IFNy secretion by decidual NK cells (95) and CD4+ Treg cells (96) affects uterine vasculature and stroma gene expression, leading to vessel instability and remodeling of decidual arteries (97) whereas TNFα may enhance apoptosis of vascular smooth muscle cells of the spiral arteries and synthesis of matrix metalloproteinases (MMPs) facilitating trophoblast invasion into the spiral arteries and placentation (98). These vascular changes are critical to ensure the increasing demands of blood flow into the uterus and placenta through pregnancy. Although unknown, it is plausible that decidual CD8+ T cells may also contribute to these processes not only through the secretion of IFNy and TNFα, but also other mediators such as IL-11, which may further favor placentation and decidualization (25, 99). In our single cell RNA sequencing analysis, cluster 3 highly expressed Gal3 and IL-32, which have both been associated with pro-angiogenic functions in tumor biology (100, 101), however whether these cells have a role in placental vascularization can only be speculated at this point. In fact, adoptive transfer of CD8+ CD122+ T cells into pregnant mice suffering from placental insufficiency significantly improved placental vascularization (72), although the intervening mechanisms require still further elucidation.

Contrasting results have been reported with regards to the production of cytolytic mediators as well as the degranulation potential of decidual CD8+ T cells. This is particularly complex when considering the CD8+ T cell differentiation states and comparisons to peripheral blood counterparts (7, 26, 34). Generally, accumulated data suggest that EM3, EMRA CD8+ T cells are important producers of GrzB in the decidua (7, 25) with more recent data pinpointing that TRM cell subset present a still higher production of GrzB. This high cytotoxic potential is in an agreement with the role of these cell subsets in host defense against infections. Noteworthy, some evidence supports that in healthy conditions cytotoxicity may be specifically reduced in distinct decidual CD8+ T cell subsets. For example, when compared to blood counterparts, stimulated decidual CD8+ naïve T cells presented lower GrzB (**Table 1**) and CD107a+ cytolytic degranulation (7, 25), and decidual EM3 and EMRA presented drastically lower basal content of GrzB and perforin (7). Suppression of cytolytic mediators may respond to the induction of tolerogenic responses in the intrauterine environment, as co-culture of TRM CD8+ T cells with trophoblasts suppressed their GrzB expression. Importantly, *in vitro* stimulation effectively upregulated the expression of perforin 1 and GrzB in the decidual CD8+ T cells, which degranulate in levels equal or higher than blood counterparts (6, 25, 26). As previously said, such retention of CD8+ T cell cytotoxic capacity might hold significant relevance for example for host defense in the case of severe infections.

Collectively, decidual CD8+ T cells, especially the EM subset, often display an exhausted profile and tissue specific features, which in homeostasis favor an intense production of cytokines (37) rather than cytolytic programs. The contribution of CD8+ T cell derived cytokines and other mediators to pregnancy processes require further empirical investigation. Decidual CD8+ T cells retain the potential for robust cytotoxicity and their dysregulation is associated with multiple pregnancy complications and thus, are critical for pregnancy success.

### Immune Surveillance and Host Defense

Through recognition and lysis of cells carrying MHC-I receptors bound to foreign peptides, CD8+ T cells are instrumental to adaptive immune responses to pathogens (102). As research on decidual CD8+ T cells have focused on their immune tolerance to fetal antigens, their function in immune surveillance and host defense has been dragged behind (103). Immune surveillance involves circulation of CD8+ T cells in the blood stream and/or lymphoid organs, trafficking into peripheral tissues as well as a localized immune surveillance in peripheral tissues. The above mentioned enrichment of clones specific for HCMV and EBV viruses (38) in HLA-A and HLA-B restricted CD8+ T cells at the feto-maternal interface support an increased capacity of decidual CD8+ T cell to fight maternal infections, although a potential role in placental infections is not clear. Extra villous trophoblast cells only express HLA-C, HLA-G and HLA-E molecules (104) and HLA-C restricted CD8+ T cells can be found in the decidua. However, questions on potential HLA-C pathogen recognition in the placenta overlap with the long-standing dilemma about the absence of inflammatory responses to allogenic HLA-C molecules in healthy conditions at the feto-maternal interface (38).

Distinct CD8+ T cell subsets possess distinct migratory patterns (11). Hence, the differentiation trajectories and functional states within the decidua are likely to influence the immune surveillance. The response of decidual CD8+ T cells to infections have been best investigated in mouse models of pregnancy affected by intracellular Listeria monocytogenes (Lm), and Lymphocytic choriomeningitis virus (LCMV). Infections with these pathogens are often associated with serious pregnancy complications, such as miscarriage, stillbirth or adverse effects for the baby (105, 106). The poor pregnancy outcomes may respond to the persistent Lm or LCMV infection of the placenta and consequent local inflammatory responses (107). Intriguingly, in the case of Lm, rather than by pathogen specific CD8+ T cells, significant immune damage was inflicted by fetal specific CD8+ T cells with an inflammatory profile accumulating at the decidua (106, 108). In turn, the clearance of pathogens in peripheral organs may be also impaired during pregnancy (106, 108, 109) suggesting that the interaction of systemic and local mechanisms might also underlie the morbidities of infection. Although in transgenic mouse models, a limited trafficking of peripheral CD8+ T cells to the decidua, e.g. due to epigenetic silencing of chemokine factors, was described (51), a CXCR3 dependent recruitment to the uterus takes place e.g. in the case of Lm infection (106, 107). Additionally, in the context of re-infection, memory CD8+ T cell proliferation was also observed in the female reproductive tract (110).

Taken together, further research on the regulation of decidual effector responses and the activation threshold would be beneficial to improve interventions for infections critical for pregnancies. The observation that maternal infections affect the pregnancy outcome by resulting in miscarriage, preterm birth, fetal malformations or enhanced risk for pregnancy complications, e.g. in the context of HCMV, ZIKV, and COVID19 infections (111–114) highlights the urgent need for further research in this field.

### Immunological Memory in Pregnancy

Maternal immune cells are exposed to foreign antigens before pregnancy: from the seminal fluid, during pregnancy: from fetal antigens, and after pregnancy: phenomenon called microchimerism (115). Seemingly, paternal-antigen specific CD8+ T cells are first primed upon encounter with the seminal fluid during coitus and are recruited into the cervix during subsequent coitus in order to prepare the uterus for and facilitate implantation (116). Whether these cells play a further role later in pregnancy and constitute the expanding decidual CD8+ T cell memory populations, and to what extent, is not known.

Immunological memory of CD8+ T cells is established upon pregnancy as fetal specific CD8+ T cells are found in the maternal circulation after pregnancy in mice and humans (24, 30, 117–119) and are suggested to play a beneficial role in subsequent pregnancies. Indeed, longer duration of paternal seminal fluid exposure reduces the risk for preeclampsia in pregnancies with that particular paternity (120, 121) and the same clonally expanded CD8+ EM cells were found in subsequent pregnancy of the same paternity (27). Furthermore, the frequency of pregnancy complications is lower in second pregnancies compared to the first pregnancy, but only with the same partner (120, 122–126). In addition to partner change, pregnancy intervals over 10 years and conception methods lacking seminal plasma priming the pregnancy (e.g. IVF) result in a higher risk of pregnancy complications, including preeclampsia (127, 128), miscarriage (129) preterm birth (130) and IUGR (131). Thus, inadequate paternal antigen-specific tolerance may be the causative factor of preeclampsia (27, 132). In mice, fetal-specific CD8+ T cells that exhibited an activated and functionally exhausted phenotype accumulated in mouse lymphoid tissues during pregnancy and in the postnatal period. These CD8+ T cells acquired a memory profile with increased expression of PD1 and Lag3 and were hyporesponsive in a second pregnancy, supporting the existence of a unique CD8+ population induced by pregnancy. Whether the circulating fetal-specific CD8+ T cells are recruited to the decidua in the second pregnancy, or whether fetal-specific CD8+ TRM cells are the primary players in the secondary response is however not clear.

## Final Remarks

**Figure 3:**
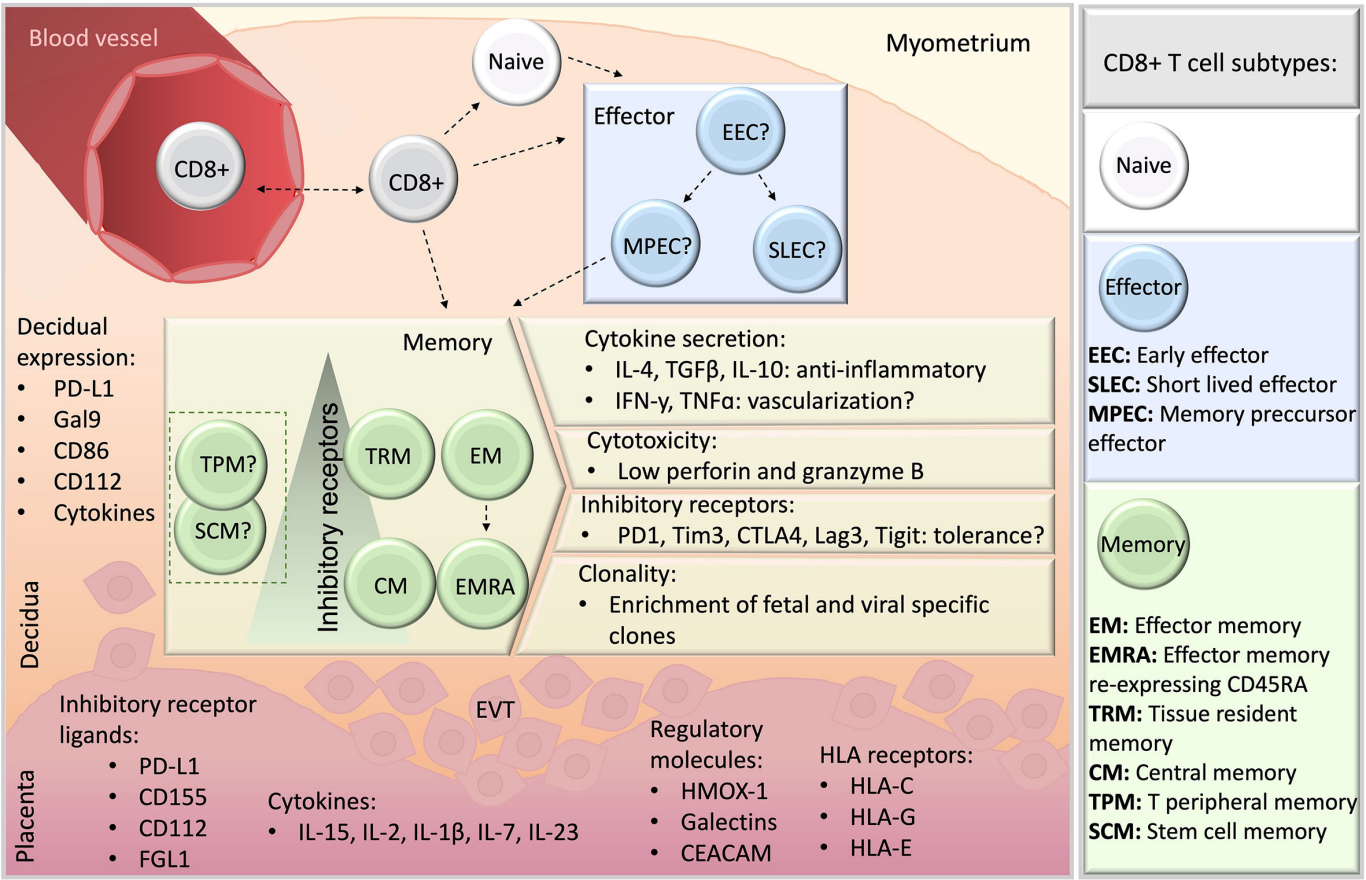
A schematic overview of CD8+ T cells at the feto-maternal interface. CD8+ T cells may migrate from blood (top, left), and whether they differentiate previously or subsequently to seeding in the decidual microenvironment remains unclear (dashed lines). Naive and memory CD8+ T cell populations have been detected in human decidua, whereas information about effector subsets is missing, indicated by a question mark. Among memory populations, TRM, EM, EMRA and CM were reported, and TPM or SCM subsets have not yet been studied. Particular features of decidual memory CD8+ T cells include unique cytokine secretion profile, elevated expression of inhibitory receptors, low basal cytotoxicity and the enrichment in clones specific for fetal antigens and viruses. Ligands for inhibitory receptors are expressed both in the decidua stroma (orange) and placenta (pink). EVT, extravillous trophoblasts.

Rather than from a widespread suppression of the immune responses, immune tolerance to fetal antigens results from a fine-tuned immune regulation. CD8+ T cell differentiation and responses are primarily influenced by the intra-uterine microenvironment. The local expression of regulatory ligands, the particular cytokine milieu (9, 24, 26, 33, 34, 37, 93), and the high levels of steroid hormones (133, 134) are critical for the establishment of immune tolerance towards the fetus (**Figure 3**). Further, the allogenic placental trophoblasts in direct contact with maternal tissues (135) may interact with maternal CD8+ T cells to upregulate their PD1 and Tim3 expression (39), whereas decidual stromal cells can promote a differentiation of peripheral CD8+CD69+ T cell into decidual TRM (34), as shown *in vitro* studies.

The independent studies here reported underscore the relevance of CD8+ T cell modulation in immune tolerance towards the fetus. The abundance of CD8+ T cells at the decidua together with their particular tissue-specific attributes portraits a heterogeneous cell compartment with varied functional features (**Figure 3**). Decidual CD8+ T cells in healthy pregnancies are cytolytic to a low extent due to low basal secretion of perforin and GrzB, despite their activated phenotype with effector and/or memory potential. Also, the potent secretory profile of decidual CD8+ T cell e.g. with regards to IFNy suggests an active response to pregnancy and a potential involvement e.g. in vascular processes in the decidua. These profiles along with the enriched expression of co-inhibitory molecules in highly differentiated cell subsets, mainly EM and TRM, appear as general features of decidual CD8+ T cells. Clearly, not only the trajectory but the functional states of decidual CD8+ T cells are tightly modulated in the intrauterine compartment, which may account as local mechanisms of tolerance towards the allogenic trophoblast. It is argued that the decidual CD8+ T cell compartment include dysfunctional, exhausted or regulatory T cells, however, a definitive characterization remains still missing. With recent advances in transcriptome analysis of single cells and in CD8+ T cell biology, overlaps of what previously was commonly accepted cellular trajectories are coming to light. This diversification is likely due to the small number of markers that can be simultaneously detected in different biological settings, such as different stages of pregnancy and pathologies, resulting in arbitrary new findings or markers and cytokines, not completely grasping the whole picture. The publicly available single cell RNA sequencing datasets on decidual immune cells include insufficient CD8+ T cells for an in-depth analysis (15). Therefore, single cell RNA sequencing tailored to CD8+ T cells, e.g. by improving cell isolation methods, including higher amounts of cells, antibody tags to identify relevant carbohydrate epitopes (CD45RA, CD57) may overcome current limitations for a better comparison to existing flow cytometry data.

Unifying their characterization and shedding light on CD8+ T cells mechanisms of pregnancy success and which components of these processes are at fault in pregnancy failure or complications, will allow to introduce new tools into current prevention strategies for pathologies such as IUGR, preeclampsia, miscarriage and preterm birth.

## Author Contributions

LH and MS wrote the manuscript. MB and ET wrote sections of the manuscript. LGl and ET performed the single cell RNA sequencing analysis. LGa, ET, and AK critically edited the manuscript. All authors contributed to the article and approved the submitted version.

## Funding

This research has been funded by Deutsche Forschungsgemeinschaft (SO1413/3). MS position and MB position are financed by Deutsche Forschungsgemeinschaft (SO1413/2) and Alexander von Humboldt-Stiftung respectively. ET is financed by Deutsche Forschungsgemeinschaft (TO235/7-2) and KFO296.

## Conflict of Interest

The authors declare that the research was conducted in the absence of any commercial or financial relationships that could be construed as a potential conflict of interest.

## Publisher’s Note

All claims expressed in this article are solely those of the authors and do not necessarily represent those of their affiliated organizations, or those of the publisher, the editors and the reviewers. Any product that may be evaluated in this article, or claim that may be made by its manufacturer, is not guaranteed or endorsed by the publisher.
