# Supplementary material for: The New Old CD8+ T Cells in the Immune Paradox of Pregnancy
Source: Front Immunol. 2021 Nov 16;12:765730. doi: 10.3389/fimmu.2021.765730 (PMC8635142; doi:10.3389/fimmu.2021.765730)
Supplement: Supplementary file 1 [file DataSheet_1.docx]

Supplementary files:

**The new old CD8+ T cells in the immune paradox of pregnancy**

Lilja Hardardottir, Maria Victoria Bazzano, Laura Glau, Luca Gattinoni, Angela Köninger, Eva Tolosa, Maria Emilia Solano

**Suppl. Table 1: Markers frequently used for identification of CD8+ T cells differentiation state**

| **Cell population** | | **Abbre-viation** | **Flow cytometry markers*** | **Known transcription factors** (1) |
| --- | --- | --- | --- | --- |
| **Naïve** | |  | **CD45RA+CCR7+** |  |
| Early effector cells | | EEC | CD127-KLRG1- |  |
| Short-lived effector cells | | SLEC | CD127-KLRG1+ |  |
| Memory precursor effector cells | | MPEC | CD127+KLRG1- |  |
| Effector memory | | EM | CD45RA-CCR7- or CD45RO+CCR7- | Tbet^hi^  Blimp1^hi^ /ID2^hi^/STAT4^hi^ |
| subsets | Effector memory 1 | EM1 | **CD27+CD28+** |  |
|  | Effector memory 2 | EM2 | **CD27+CD28-** |  |
|  | Effector memory 3 | EM3 | **CD27-CD28-** |  |
|  | Effector memory 4 | EM4 | **CD27-CD28+** |  |
| Effector memory re-expressing CD45RA | | EMRA | **CD45RA-CCR7-** |  |
| Tissue resident memory | | TRM | **CD103+CD69+** | Tbet^low^ / TCF1^low^ / Hobit ^hi^ / Blimp1^hi^ /KLF2^-/low^ / Eomes ^-/low^ |
| Central memory | | CM | CD45RA-CCR7+ | Tbet^low^ / Eomes ^hi^ / TCF1^hi^ / BCL6 ^hi^ / STAT3 ^hi^ / ID3 ^hi^ |
| Stem cell memory | | SCM | CD45RA+CCR7+ CD27+ CD95+ | TCF1^hi^ |
| Peripheral memory | | TPM | CX3CR1^int^ CD27^hi^CD127^hi^ |  |

(*) Refers to markers used to identify the respective population by Flow cytometry. The markers reported in the context of research in decidua tissues are highlighted in **bold**.

| **Cell population (Abbrev.)** | **Trimesters** | | **Cytokine secretion** | **Cytoxicity** | **Inhibitory molecules** | **Pathology** |
| --- | --- | --- | --- | --- | --- | --- |
|  | **1** | **3** |  |  |  |  |
| Naïve | - **↓** (2–6) | -/+  **↓** (4,5,7–9) | -/+ IFNy  - TNFa  (4) | PRF_low_* GZMB _low_ * -CD107a **↓** (4,7) | +PD1^+^Tim3^+^  (3) | N/D |

**Supplementary Table 2. CD8+ naive cells in human decidua**

The data is based on published flow cytometry analysis using markers CD45RA and CCR7 as well as inhibitory markers and cytokine expression Black symbols **↑**, **↓,** and **=** indicate significantly higher, lower or not significantly different compared to CD8+ T cells in peripheral blood. **-** very low (0-5%); + low: 6-20%; ++ high: 21-55 %; +++ very high >56 % of the reference population. The symbol “/” represents diverging values. N/D: data not determined. Low in subscript refers to a low mean fluorescence intensity by flow cytometry. Cytokine secretion/cytoxicity was measured following PMA/Iomycin stimulation except those marked with „*“. PRF: perforin-1; GrzB: granzyme B.

**Suppl. Table 3. CD8 T cell inhibitory receptors and their ligands at the feto-maternal interface**.

| **Inhibitory  Receptor** | | **PD1** | **Tim3** | **CTLA4** | | **LAG3** | | **Tigit** | |
| --- | --- | --- | --- | --- | --- | --- | --- | --- | --- |
| Ligands | | PD-L1 | Gal9 | CD80 | CD86 | HLA class II/ MHC-II | FGL1 | CD155 | CD112 |
| Feto-maternal interface | Ligand-positive cells | TB, MΦ | DC, GC  MΦ, NK,  HB, ENC | MO | MΦ, DC  MC, HB | MΦ, DC,  MC | HB | TB | MΦ, SC, ENC, EPC  HB, TB |
|  | References in decidua | (10) (11) | (3) | (12) | | (13) | N/D | N/D | |

TB: Trophoblasts, MΦ: Macrophages, SC: Stromal cells, DC: dendritic cells, GC: granulocytes, NK: NK cells, HB: Hofbauer cells, ENC: endothelial cells. Monocytes: MC, EPC: epithelial cells, N/D: not determined. Expression of ligand positive cells is based on online published single cell sequencing dataset (14).

**
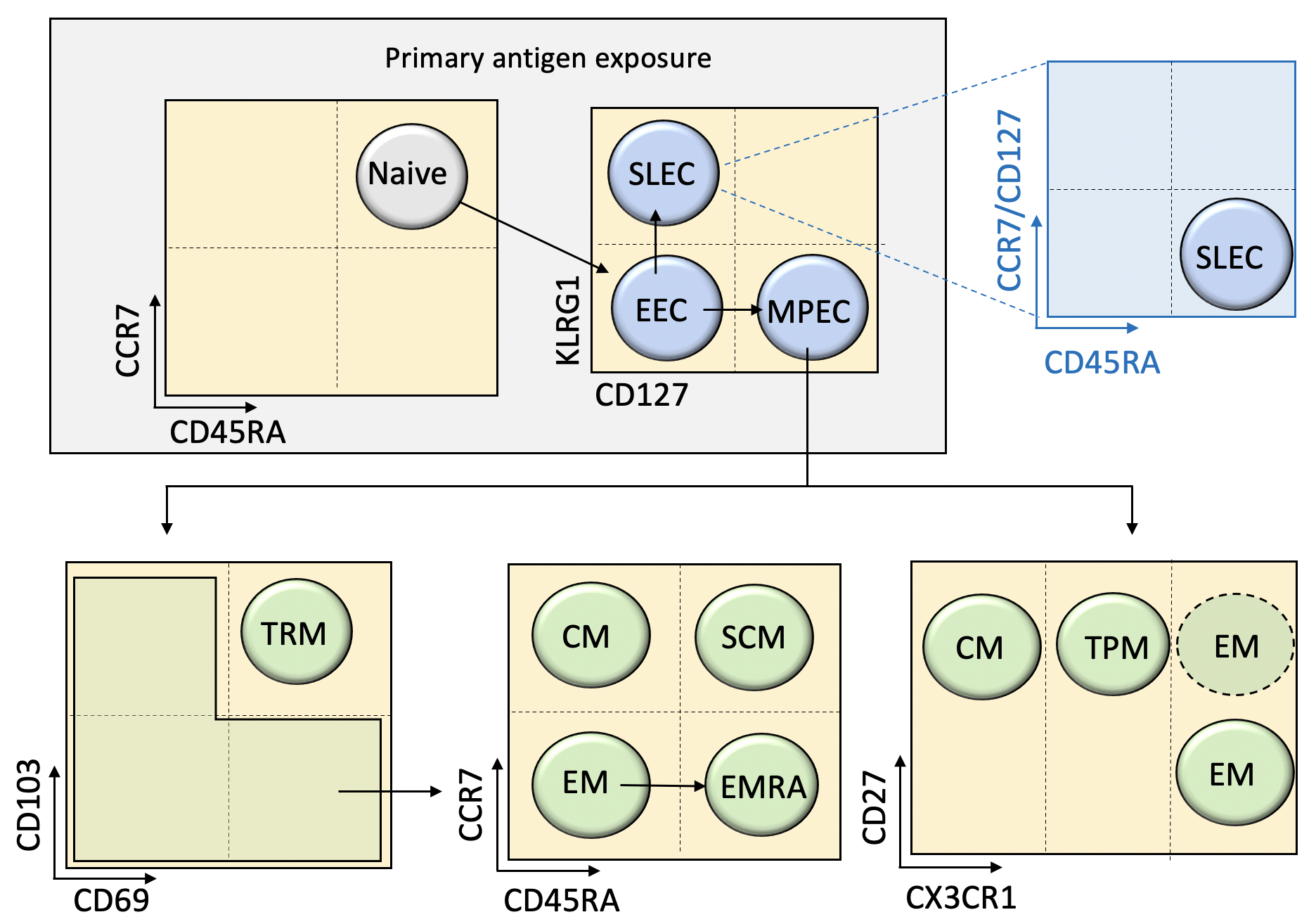
**

**Suppl. Figure 1. CD8+ T cell differentiation.** Graphical representation of differenciation trajectories according to the „linear model“ as well as markers used to identify each CD8+ T cell subset as named in Suppl. Table 1. The arrows denote the differenciation process and not a gating strategy.


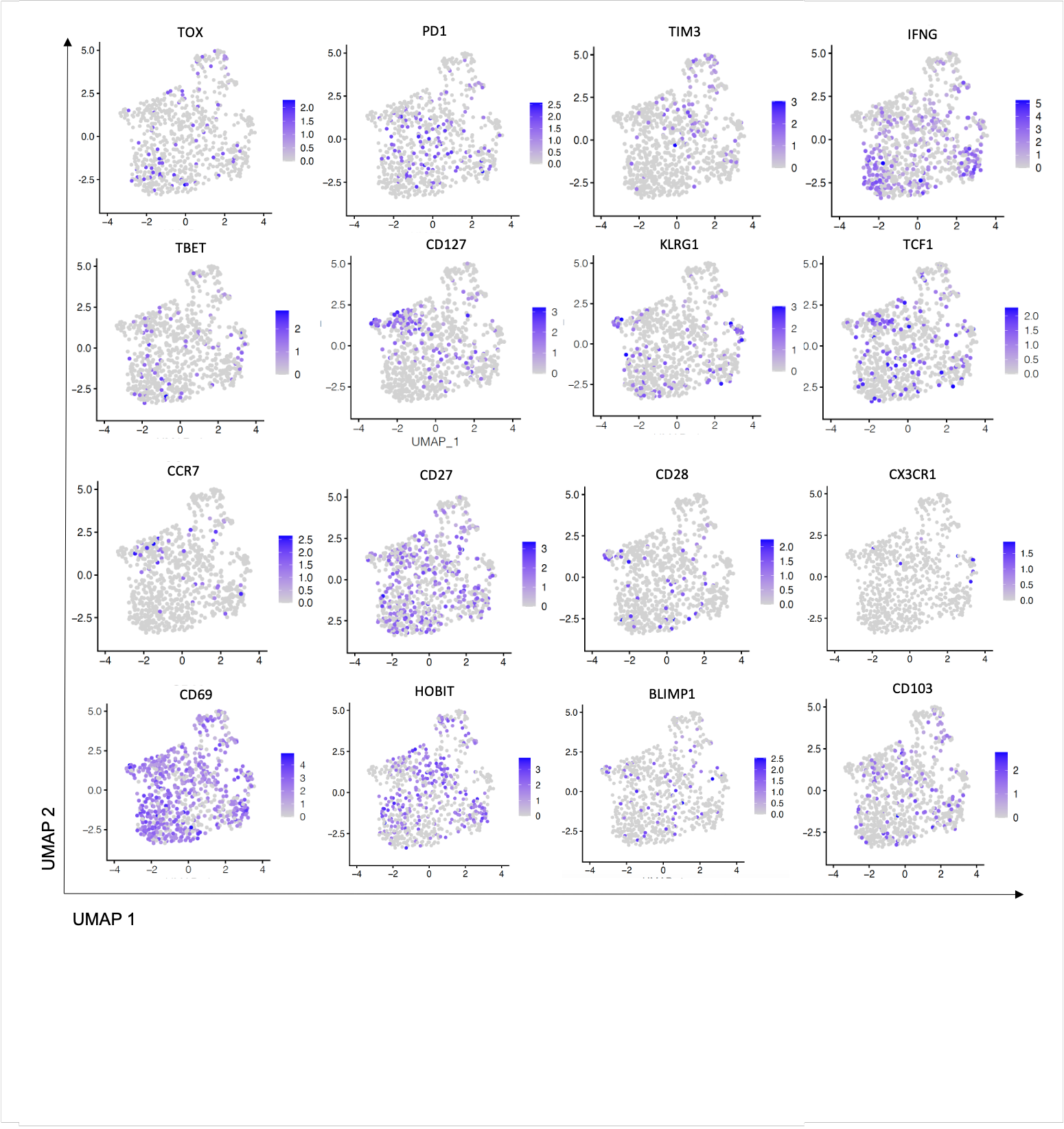


**Suppl. figure 2.** Heatmaps of selected markers from single cell gene expression analysis (15). Protein names are used for simplification.

**Supplementary methodological information**

Selected keywords were used for the literature search in Pubmed. Articles were not excluded based on publication date. Articles for constuction of table 1 (characterization of human decidual CD8+ T cells) were limited to flow cytometry data using CD45RA/CD45RO and CCR7 enabling direct comparison.

Publicly available single-cell RNA sequencing dataset (GEO:GSE164449, <https://www.ncbi.nlm.nih.gov/geo/>) from the decidua of three healthy first trimester pregnancy samples (on average 7 weeks of gestation) (15) was re-analysed using the R-package Seurat (16). Following data cleaning and selection of MS4A7-CD3E+CD8A+ cells, UMAP dimensionality reduction was performed and cell clustering and identification of differentially expressed genes per cluster was conducted.

The decidual tissues were enzymatically digested and erythrocytes were lysed. Sorted CD45+ living cells were processed with the Chromium Single Cell 3´Reagent kits and libraries were generated with an approach by 10x Genomics. Libraries were sequenced on Illumina NovaSeq 6000 by Novogene Bioinformatics Technology Co. (15).

**References**

1. Martin MD, Badovinac VP. Defining Memory CD8 T Cell. (2018) 9:1–10. doi:10.3389/fimmu.2018.02692

2. Liu L, Huang X, Xu C, Chen C, Zhao W, Li D, Li L, Wang L. Decidual ­ CD8 + T cells exhibit both residency and tolerance signatures modulated by decidual stromal cells. *J Transl Med* (2020)1–14. doi:10.1186/s12967-020-02371-3

3. Wang SC, Li YH, Piao HL, Hong XW, Zhang D, Xu YY, Tao Y, Wang Y, Yuan MM, Li DJ, et al. PD-1 and Tim-3 pathways are associated with regulatory CD8+ T-cell function in decidua and maintenance of normal pregnancy. *Cell Death Dis* (2015) 6:1–10. doi:10.1038/cddis.2015.112

4. van der Zwan A, Bi K, Norwitz ER, Crespo ÂC, Claas FHJ, Strominger JL, Tilburgs T. Mixed signature of activation and dysfunction allows human decidual CD8 + T cells to provide both tolerance and immunity. *Proc Natl Acad Sci* (2018) 115:201713957. doi:10.1073/pnas.1713957115

5. Morita K, Tsuda S, Kobayashi E, Hamana H, Tsuda K. Analysis of TCR Repertoire and PD-1 Expression in Decidual and Peripheral CD8 + T Cells Reveals Distinct Immune Mechanisms in Miscarriage and Preeclampsia. (2020) 11: doi:10.3389/fimmu.2020.01082

6. Zeng W, Liu X, Liu Z, Zheng Y, Yu T, Fu S, Li X, Zhang J, Zhang S, Ma X, et al. Deep surveying of the transcriptional and alternative splicing signatures for decidual CD8+T cells at the first trimester of human healthy pregnancy. *Front Immunol* (2018) 9:1–16. doi:10.3389/fimmu.2018.00937

7. Tilburgs T, Schonkeren D, Eikmans M, Nagtzaam NM, Datema G, Swings GM, Prins F, Lith JM Van, Mast BJ Van Der, Roelen L, et al. Human Decidual Tissue Contains Differentiated CD8 + Effector-Memory T Cells with Unique Properties. (2010) doi:10.4049/jimmunol.0903597

8. Powell RM, Lissauer D, Tamblyn J, Beggs A, Cox P, Moss P, Kilby MD. Decidual T cells exhibit a highly differentiated phenotype and demonstrate potential fetal-specificity and a strong transcriptional response to interferon. (2017) 199:3406–3417. doi:10.4049/jimmunol.1700114.Decidual

9. Feyaerts D, Benner M, Cranenbroek B Van, Heijden OWH Van Der. Human uterine lymphocytes acquire a more experienced and tolerogenic phenotype during pregnancy. (2017)1–10. doi:10.1038/s41598-017-03191-0

10. Veras E, Kurman RJ, Wang TL, Shih IM. PD-L1 expression in human placentas and gestational trophoblastic diseases. *Int J Gynecol Pathol* (2017) 36:146–153. doi:10.1097/PGP.0000000000000305

11. Barton BM, Xu R, Wherry EJ, Porrett PM. Pregnancy promotes tolerance to future offspring by programming selective dysfunction in long-lived maternal T cells. *J Leukoc Biol* (2016) 101:975–987. doi:10.1189/jlb.1a0316-135r

12. Wang S, Sun F, Li M, Qian J, Chen C, Wang M, Zang X, Li D, Yu M, Du M. The appropriate frequency and function of decidual Tim-3+CTLA-4+CD8+ T cells are important in maintaining normal pregnancy. *Cell Death Dis* (2019) 10: doi:10.1038/s41419-019-1642-x

13. YH Z, HX S. Immune checkpoint molecules in pregnancy: Focus on regulatory T cells. *Eur J Immunol* (2020) 50:160–169. doi:10.1002/EJI.201948382

14. Vento-tormo R, Efremova M, Botting RA, Turco MY, Vento-tormo M, Meyer KB, Park J, Stephenson E, Polański K, Goncalves A, et al. Single-cell reconstruction of the early maternal – fetal interface in humans. (2018) doi:10.1038/s41586-018-0698-6

15. Chen P, Zhou L, Chen J, Lu Y, Cao C, Lv S, Wei Z, Wang L, Chen J, Hu X, et al. The Immune Atlas of Human Deciduas With Unexplained Recurrent Pregnancy Loss. *Front Immunol* (2021) 12:1–16. doi:10.3389/fimmu.2021.689019

16. Stuart T, Butler A, Hoffman P, Hafemeister C, Papalexi E, Mauck WM 3rd, Hao Y, Stoeckius M, Smibert P, Satija R. Comprehensive Integration of Single-Cell Data. *Cell* (2019) 177:1888-1902.e21. doi:10.1016/j.cell.2019.05.031
